# Protective effects of various ratios of DHA/EPA supplementation on high-fat diet-induced liver damage in mice

**DOI:** 10.1186/s12944-017-0461-2

**Published:** 2017-03-29

**Authors:** Tingting Shang, Liang Liu, Jia Zhou, Mingzhen Zhang, Qinling Hu, Min Fang, Yongning Wu, Ping Yao, Zhiyong Gong

**Affiliations:** 1 College of Food Science and Engineering, Wuhan Polytechnic University, 68 XueFuNan Road, Wuhan, 430023 People’s Republic of China; 2 China National Center For Food Safety Risk Assessment, Beijing, 100022 China; 3 Department of Nutrition and Food Hygiene, School of Public Health, Tongji Medical College, Huazhong University of Science and Technology, Wuhan, 430030 People’s Republic of China

**Keywords:** N-3 PUFA, DHA/EPA, Lipid metabolism, Liver damage, Fra1, C-Jun, C-Fos

## Abstract

**Background:**

A sedentary lifestyle and poor diet are risk factors for the progression of non-alcoholic fatty liver disease. However, the pathogenesis of hepatic lipid accumulation is not completely understood. Therefore, the present study explored the effects of dietary supplementation of various ratios of docosahexaenoic acid (DHA) and eicosapentaenoic acid (EPA) on a high-fat diet-induced lipid metabolism disorder and the concurrent liver damage.

**Methods:**

Using high-fat diet-fed C57BL/6 J mice as the animal model, diets of various ratios of DHA/EPA (2:1, 1:1, and 1:2) with an n-6/n-3 ratio of 4:1 were prepared using fish and algae oils enriched in DHA and/or EPA and sunflower seed oils to a small extent instead of the high-fat diet.

**Results:**

Significantly decreased hepatic lipid deposition, body weight, serum lipid profile, inflammatory reactions, lipid peroxidation, and expression of adipogenesis-related proteins and inflammatory factors were observed for mice that were on a diet supplemented with DHA/EPA compared to those in the high-fat control group. The DHA/EPA 1:2 group showed lower serum triglycerides (TG), total cholesterol (TC), and low-density lipoprotein-cholesterol levels, lower SREBP-1C, FAS, and ACC-1 relative mRNA expression, and higher Fra1 mRNA expression, with higher relative mRNA expression of enzymes such as AMPK, PPARα, and HSL observed in the DHA/EPA 1:1 group. Lower liver TC and TG levels and higher superoxide dismutase levels were found in the DHA/EPA 2:1 group. Nonetheless, no other notable effects were observed on the biomarkers mentioned above in the groups treated with DHA/EPA compared with the DHA group.

**Conclusions:**

The results showed that supplementation with a lower DHA/EPA ratio seems to be more effective at alleviating high-fat diet-induced liver damage in mice, and a DHA/EPA ratio of 1:2 mitigated inflammatory risk factors. These effects of n-3 polyunsaturated fatty acids (PUFA) on lipid metabolism may be linked to the upregulation of Fra1 and attenuated activity of c-Jun and c-Fos, thus ultimately reducing the severity of the lipid metabolism disorder and liver damage to some extent.

## Background

An unhealthy lifestyle involving poor dietary habits and low physical activity is a risk factor for the progression of non-alcoholic fatty liver disease (NAFLD) [1]. Although it is generally believed that nutrient modulation is a positive means to alleviate this problem [2] since the type and quantity of fat consumed regulate hepatic lipid composition and gene expression [3], the pathogenesis of hepatic lipid accumulation is not completely understood [4].

Decreasing dietary saturated fatty acid (SFA) intake [5] and replacing it with polyunsaturated fatty acids (PUFA) [6] has been advocated as the main dietary strategy to prevent NAFLD caused by anti-inflammatory, antioxidative, and hypolipidemic effects of PUFAs. Meanwhile, long-chain PUFAs, mainly n-6 PUFA and n-3 PUFA, especially eicosapentaenoic (C20:5 n-3, EPA) and docosahexaenoic (C22:6 n-3, DHA) acids, have frequently been reported to influence NAFLD risk factors [7]. Reduced hepatic lipid content with concomitant antioxidant and anti-inflammatory responses favoring insulin sensitivity in mice with high-fat diet-induced obesity are ascribed to the repletion of liver n-3 PUFA levels by n-3 PUFA dietary supplementation [7]. Nevertheless, evidence suggests that selectively increasing the n-3 PUFA proportion of the total PUFA intake [8, 9] has more significant inhibitory effects on inflammation and NAFLD than simply increasing n-3 PUFA intake [10]. Interestingly, several studies have also demonstrated the latent effects of the dietary proportion of n-6/n-3 on liver damage [10, 11], and a low n-6/n-3 ratio was shown to be more effective for the prevention of diet- or obesity-induced fatty liver than that of a high n-6/n-3 ratio. Consequently, a reasonable proportion of n-6/n-3 should be taken into consideration when investigating the effects of PUFAs on NAFLD [10].

Recently, there has been an increasing interest in the effects of various ratios of DHA and EPA on chronic diseases in rat models [12–15], and studies conducted to evaluate these effects suggest that both n-6/n-3 ratios and DHA/EPA ratios should be considered simultaneously. Additionally, our research group has previously reported that DHA and EPA from fish and algal oils are more beneficial than plant oils enriched in α-linolenic acid (C18:3 n-3, ALA) at delaying atherosclerosis progression in model *apoE* ^*−/−*^ mice; an intervention with higher DHA/EPA ratios was found to be beneficial for reducing high cholesterol concentrations in the blood [16]. Nevertheless, DHA and EPA differ in their physiological functions, and a new study indicated that DHA supplementation leads to a greater reduction in specific markers of inflammation than that by an equal dose of EPA [17]. In view of these observations, it remains unclear whether DHA is more effective than DHA/EPA, there is an optimal ratio of DHA and EPA supplementation for reduction of high-fat diet-induced liver damage, and regularizing the ratio of DHA and EPA has differential effects on various symptoms.

Therefore, in the present study, we sought to investigate the role of dietary interventions with various ratios of DHA/EPA (2:1, 1:1, and 1:2) in high-fat diet-induced liver damage at an n-6/n-3 ratio of 4:1, which is optimal for human health as recommended by the World Health Organization (WHO). Healthy C57BL/6 J mice were employed as animal models. Mice were fed high-fat diets supplemented with sunflower seed, algae, and fish oils containing different DHA/EPA ratios (2:1, 1:1, and 1:2).

## Methods

### Animals and diets

Ninety healthy male C57BL/6 J mice (age, 5 weeks; weight, 18 ± 2 g) were supplied by Hubei Research Center of Laboratory Animals (Wuhan, China) with animal license SCXK (Hubei) 2008–0005. Animal experiments described in this study were conducted with the approval of the Tongji Medical College Council on Animal Care Committee and in accordance with the Guiding Principles of the Care and Use of Laboratory Animals published by the US National Institutes of Health. Before the mice were randomized into six groups (*n* = 15), they were acclimated to the laboratory for 7 days with basic feed, which was purchased from WQJX Bio-technology (Wuhan, China). High-fat diet I (HFD I) was composed of 700 g of basic feed, 150 g of lard, 100 g of yolk powder, 40 g of casein, and 10 g of cholesterol per kg. In high-fat diet II (HFD II), the 150 g/kg lard was reduced to 100 g/kg. The normal control group (NC) was fed with basic feed; the high-fat control group (HFC) was fed with HFD I; the DHA group received HFD II plus algal oils (containing 100 mg of DHA per 260 mg) and sunflower seed oil (containing n-6 PUFA at 60% but low amount of n-3 PUFA, adjusting the proportion of n-6/n-3 to 4:1); and the DHA/EPA group was fed HFD II as well as fish oils (containing 200 mg DHA and 400 mg EPA per 1900 mg) and algal oils to achieve various DHA/EPA ratios (2:1, 1:1, and 1:2), and sunflower seed oils to adjust the n-6/n-3 ratio to 4:1. The total caloric intakes of mice in all groups were equivalent except for mice in the NC group. The fatty acid compositions of oils, basic feed, control diet, and high fat diets are described in Table 1. The PUFAs were administered orally (0.75 mg/g body weight) every day for 11 weeks, and NC as well as HFC mice were given the same dose of distilled water via intragastric administration as control groups. The mice were individually housed in a humidity- and temperature-controlled room (relative humidity 60% and temperature 20–22 °C) with a 12-h light/dark cycle environment and were provided ad libitum access to water and their group-specific diet. Measurements of body weight and food intake were taken weekly. After 11 weeks, five mice from each group were anesthetized with isoflurane, and blood was drawn through the retro-orbital sinus puncture prior to killing the mice by decapitation after an overnight fast and used for analyses to ensure the establishment of models of steatosis and liver lesions. The rest of the mice were euthanized in the same way. Serum was collected from blood, subsequently centrifuged (4000×*g*, 4 °C, 10 min) after incubation at room temperature for 2 h, and then stored at −80 °C until analysis. Fresh liver tissue samples were collected for histopathology analysis or were rapidly frozen and stored at −80 °C before use.

**Table 1:** Fatty acid composition of oils and mixed diets supplemented to mice

Fatty acid	mg per 100 mg total fatty acid									
	Sunflower seed oil	Fish oil	Algae oil	Basic feed	HFD I	HFD II	DHA	DHA/EPA 2:1	DHA/EPA 1:1	DHA/EPA 1:2
C14:0	0.1	0.3	8.7	0	0	0	2.6	1.4	0.8	0.1
C14:1	0	0.2	0.3	0	0	0	0.1	0.1	0.1	0.1
C15:0	0	0.5	0.2	0	0	0	0.1	0.1	0.1	0.1
C16:0	6.5	10.1	15.8	27.0	42.0	43.7	9.6	8.6	8.1	8
C16:1	0.1	4.7	3.5	0	1.8	1.7	1.1	1.1	1.2	1.5
C17:0	0	0.1	0.3	0	0	0	0.1	0.1	0.1	0
C17:1	0	0.1	0	0	0	0	0.0	0	0	0
C18:0	5.2	2.8	0	4.7	0.2	0.3	3.9	4.4	4.6	4.9
C18:1	24.9	7.9	13.5	21.2	16.5	15.6	22.8	22.2	22.4	22.2
C18:2(LA)n-6	60.7	1.9	1.6	36.9	21.5	21.7	46.4	48.4	49.3	49.9
C18:3(ALA)n-3	0	2	0	0	16.0	15.3	0.0	0.2	0.4	0.2
C20:1	0	0.1	0.3	0	0.13	0.12	0.1	0.1	0.1	0
C20:2	0	0.2	0	0	0	0	0.0	0	0	0.1
C20:4(AA)n-6	0	1.5	0.5	0.5	0.19	0.20	0.1	0.2	0.3	0.4
C20:5(EPA)n-3	0.2	35.1	0	0	0	0	0.2	4.3	6.2	8.1
C22:1	0	0.3	3.5	0	0	0	1.0	0.5	0.3	0.1
C22:5(DPA)n-3	0	2.4	0	0	0	0	0.0	0.3	0.4	0.6
C22:6(DHA)n-3	0	18.2	40.1	0	0	0	11.8	8	6	4.3
Total n-6	60.7	3.4	2.1	36.9	21.5	21.7	46.6	48.6	49.6	50.3
Total n-3	0.2	57.7	40.1	0	16.0	15.3	12.0	12.8	13	13.2
n6/n3					1.3	1.4	3.9	3.9	3.8	3.8
DHA/EPA	0	1.9	0	0	0	0	59.0	1.9	1	0.5

### Analysis of steatosis and liver lesions

The liver was removed and weighed immediately after the mice were euthanized. Liver fragments were sectioned at a thickness of 5 μm, and fixed with 4% paraformaldehyde overnight. Then the slices were stained with oil red O, in order to analyze the steatosis and liver lesions. Images were acquired by means of an inverted fluorescence microscope (Olympus Co., Japan) equipped with a SONY DXC-970MD color video camera, and analyzed using the Image-Pro plus program.

### Measurement of serum and liver lipid profiles

The serum triglyceride (TG), total cholesterol (TC), high-density lipoprotein-cholesterol (HDL-C), and low-density lipoprotein-cholesterol (LDL-C) levels were measured using similar commercial kits (Biosino Biotechnology Co., Ltd., Beijing, China) with spectrophotometric methods in accordance with the manufacturer’s instructions.

To determine the hepatic TC and TG levels, 50 mg of frozen liver tissue was placed in a test tube with 450 μl of isopropanol. The homogenate was incubated at 4 °C for 36 h and then centrifuged at 4000×*g* and 4 °C for 10 min, and the supernatant was used for analyses by means of the corresponding commercial kits (Biosino Biotechnology Co., Ltd., Beijing, China) with spectrophotometric methods in accordance with the manufacturer’s instructions.

### Measurement of serum ALT, AST, GSH, SOD, MDA, and inflammatory cytokines, and liver GSH, SOD, and MDA

The serum alanine aminotransferase (ALT), aspartate aminotransferase (AST), glutathione (GSH), superoxide dismutase (SOD), and malondialdehyde (MDA) were measured by means of similar commercial kits (Nanjing Jiancheng Corporation, Nanjing, China) in accordance with the manufacturer’s recommendations. Serum tumor necrosis factor-alpha (TNF-α), interleukin-1β (IL-1β), and interleukin-6 (IL-6) were determined using the corresponding commercial kits (Cloud-Clone Corp, Wuhan, China) by ELISA methods according to the manufacturer’s instructions.

To analyze the hepatic levels of GSH, SOD, and MDA, 50 mg of liver tissues were homogenized with 450 μl of normal saline. After centrifugation at 4000×*g* and 4 °C for 10 min, the supernatant of the homogenate was used for analysis with the corresponding commercial kits (Nanjing Jiancheng Corporation) as specified by the manufacturer. In addition, protein content was measured using commercial bicinchoninic acid (BCA) kits (Cloud-Clone Corp, Wuhan, China).

### Real-time PCR

The total RNA was extracted from liver tissues using the RNAiso Plus reagents (TaKaRa BIO Inc., Dalian, China). Then, the cDNA was extracted using a Prime Script RT Reagent Kit (TaKaRa BIO Inc.). The mRNA was quantified with a real-time PCR machine (IQ5, Bio-Rad, USA) using SYBR Premix Ex Taq (TaKaRa BIO Inc., Dalian, China) and specific primers (BGI Tech Solutions Co., Ltd., Shenzhen, China) in accordance with the manufacturer’s recommendations. The forward and reverse primers for the target genes are listed in Table 2. Relative gene expression was normalized (by means of the C_T_ method) to that of the endogenous control β-actin, and the final results were calculated using the formula 2^−ΔΔCt^.

**Table 2:** Real-time quantitative PCR primer sequences

Gene	Forward primer 5′–3′	Reverse primer 5′–3′
AMPK	GCCATGCGCAGACTAGTT	AGGATGTATGGCCGATCTTC
SREBP-1	GTGAGCCTGACAAGCAATCA	GGTGCCTACAGAGCAAGAGG
PPARγ	TTTCAAGGGTGCCAGTTT	GAGGCCAGCATCGTGTAG
PPARα	CCTCAGGGTACCACTACGGAGT	GCCGAATAGTTCGCCGAA
ACC-l	TACTGAACTACATCTTCTCCC	AAGCAATAAGAACCTGACGA
FAS	CAAATACAATGGCACCCTGA	TGGCGAAGCCGTAGTTAGTT
SCD-1	GAATTCATGCCTGCGCACTTGCTACA	CTCGAGTCAGCCGCTCTTGTGACTCC
CPT-1	TTAACAGCAACTACTACGCC	CCAGAAGACGAATAGGTTTGAG
HSL	GCCACAATGACACAGTCACTGGT	CAGGCAGCGGCCGTAGAAGCA
ACOX	GGTGGCTGTTAAGAAGAGTG	AAGATGAGTTCCATGACCCA
c-Jun	ATGACTGCAAAGATGGAAACG	AAGTTGCTGAGGTTGGCG
c-Fos	TACTACCATTCCCCAGCC	GCTCTACTTTGCCCCTTC
Fra1	TCATCTGGAGAGGTGGGTCC	CTGCGGTTCTGACTCACTCG
TNF-α	AGCCCCCAGTGTGTATCCTT	ACAGTCCAGGTCACTGTCCC
IL-1β	CTTCAGGCAGGCAGTATCACTC	TTGTTGTTCATCTCGGAGCC
IL-6	AGTTGCCTTCTTGGGACTGA	TCCACGATTTCCCAGAGAAC
β-actin	TTCGTTGCCGGTCCACACCC	GCTTTGCACATGCCGGAGCC

### Analysis of western blots

Protein expression levels of c-Jun, c-Fos, and Fra1 were analyzed by western blotting. Briefly, the total protein was extracted in PMSF:RIPA = 1:99 (*v*/v), a homogenizing and lysis buffer. Equal amounts of protein extracts, which were normalized to the total amount of protein, were mixed (1:3, *v*/v) with the loading buffer for electrophoresis using 10% SDS-PAGE gels kits (Boster Bio-engineering, Ltd., Wuhan, China). The mixture was subsequently electroblotted to a nitrocellulose transfer membrane (Millipore, USA) by a Trans-Blot SD semi-dry transfer cell (Bio-Rad, USA) as per the manufacturer’s instructions. The target protein levels were detected with specific primary antibodies (Bioss Biological Technology, Ltd., Wuhan, China) against the target protein, and then incubated with the species-specific secondary antibody, horseradish peroxidase-conjugated anti-rabbit IgG antibody (Boster Bio-engineering), as recommended by the manufacturer. The chemiluminescence intensity of the bands was quantified by an ECL developer (Millipore, USA) using a Western Blotting Detection System (Bio-Rad, USA), and the optical densities of the bands were detected using Image Lab 5.1 software (Bio-Rad). Data were corrected to eliminate background noise and were standardized to the optical density (OD/mm^2^) of β-tubulin (Boster Bio-engineering).

### Statistical analysis

All data were recorded with Excel. After confirming the homogeneity of variances, differences among the groups were tested by one-way ANOVA. Means ± SEM were calculated for each group, and the figures were prepared using GraphPad Prism v6.0.1 software. The significant differences were shown by the LSD multiple comparison test using SPSS statistics 17.0 software, and any two groups without the same lowercase and uppercase letters, as marked in the figures, indicate a significant difference of *P* value <0.05 and <0.01, respectively.

## Results

### Effects of various ratios of DHA/EPA with an n-6/n-3 ratio of 4:1 on steatosis and liver cell histopathological lesions

**Fig. 1:**
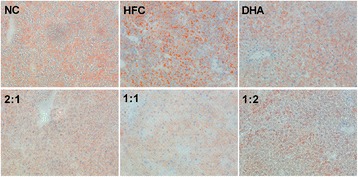
Effects of DHA/EPA on steatosis and liver cell histopathological lesions in high-fat diet-induced mice. Slices of liver tissues stained with oil red O (*n* = 5, ×200)

At 12 weeks post-administration, the histopathological lesions of livers in all groups were assessed. The oil red O staining patterns showed orange lipid droplets and blue nuclei, which were smaller and fewer in number in the DHA/EPA (2:1, 1:1, and 1:2) groups; while obvious numerous orange-red lipid droplets were observed in HFC-treated mice, nearly none were observed in the NC group (Fig. 1). Moreover, fewer lipid droplets were observed in mouse groups treated with DHA/EPA (ratios of 2:1, 1:1, and 1:2) compared to the group treated with DHA.

### Effects of various ratios of DHA/EPA with an n-6/n-3 ratio of 4:1 on body weight, serum, and liver lipids

**Fig. 2:**
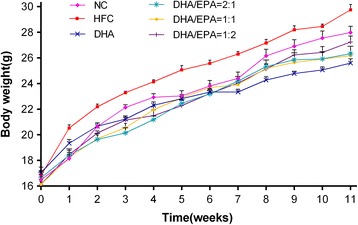
Effects of various ratios of DHA/EPA on growth of high-fat diet-induced mice. Each value is a mean ± SEM (*n* = 10)

Compared to the HFC group, significant decreases (*p* < 0.05) in serum TC, TG, LDL-C levels, and liver TC and TG levels were observed in 12-week n-3 PUFA supplementation groups (Table 3). The growth curve of mice revealed that the body weight increased slower with n-3 PUFA supplementation than with HFC treatment (Fig. 2), indicating the inhibitory effect of n-3 PUFA supplementation with DHA and different DHA/EPA ratios (2:1, 1:1, and 1:2) in the mice. Moreover, mice treated with a DHA/EPA ratio of 2:1 and 1:2 showed a reduced hepatic organ coefficient with a significant difference of *p* < 0.01, while the statistical differences of reduction in DHA and DHA/EPA 1:1 groups were *p* < 0.05 compared to those in the HFC group (Table 3). Mice treated with a DHA/EPA ratio of 1:2 showed a more appreciable decrease in serum TC, TG, and LDL-C levels, whereas mice treated with the DHA/EPA ratio of 2:1 showed lowered liver TC and TG levels to a greater degree. However, mice treated with various ratios of DHA/EPA (2:1, 1:1, and 1:2) revealed no reduction in serum TC, TG, or LDL-C levels or liver TC and TG levels in comparison with those in the DHA group. Nevertheless, as compared to the levels in the HFC group, serum HDL-C levels in DHA and DHA/EPA (2:1, 1:1, and 1:2) groups were significantly higher (*p* < 0.01). Furthermore, serum HDL-C levels increased more significantly in the DHA/EPA 1:2 group than in other DHA/EPA groups (*p* < 0.01), but the increase was not remarkable in comparison to that in the DHA group.

**Table 3:** Effects of various ratios of DHA/EPA on serum and liver lipids in high-fat diet-induced mice

	NC	HFC	DHA	DHA/EPA 2:1	DHA/EPA 1:1	DHA/EPA 1:2
Serum TC/(mmol/L)	3.693 ± 0.298^aA^	5.175 ± 0.368^bB^	3.559 ± 0.246^aA^	4.144 ± 0.323^aAB^	4.058 ± 0.181^aA^	3.935 ± 0.180^aA^
Serum TG/(mmol/L)	1.198 ± 0.045^bB^	1.388 ± 0.029^cC^	0.862 ± 0.035^aA^	0.981 ± 0.060^aA^	0.986 ± 0.028^aA^	0.879 ± 0.046^aA^
Serum HDL-C/(mmol/L)	1.144 ± 0.044^cdBC^	0.791 ± 0.028^aA^	1.270 ± 0.043^dC^	1.015 ± 0.017^bB^	1.128 ± 0.038^bcBC^	1.267 ± 0.029^dC^
Serum LDL-C/(mmol/L)	1.954 ± 0.082^bcB^	2.654 ± 0.055^dC^	1.600 ± 0.081^aA^	2.158 ± 0.087^cB^	2.070 ± 0.076^bcB^	1.914 ± 0.067^bB^
Liver TC/(mmol/L)	1.276 ± 0.099^aA^	2.452 ± 0.101^dD^	1.515 ± 0.078^bAB^	1.689 ± 0.055^bcBC^	1.811 ± 0.055^cBC^	1.823 ± 0.038^cC^
Liver TG/(mmol/L)	3.559 ± 0.184^bAB^	6.177 ± 0.330^dD^	2.693 ± 0.166^aA^	4.293 ± 0.100^cBC^	4.494 ± 0.350^cC^	4.538 ± 0.172^cC^
hepatic organ coefficient /(g/100 g)	3.835 ± 0.098^aA^	4.268 ± 0.037^bB^	4.019 ± 0.080^aAB^	3.934 ± 0.097^aA^	4.033 ± 0.037^aAB^	3.939 ± 0.060^aA^

Note: Each value is the mean ± SEM (*n* = 8), and any two groups without the same lowercase or uppercase letters, as marked in the table, indicate a significant difference of *p* < 0.05 and *p* < 0.01, respectively

### Effects of various ratios of DHA/EPA with an n-6/n-3 ratio of 4:1 on liver damage

**Fig. 3:**
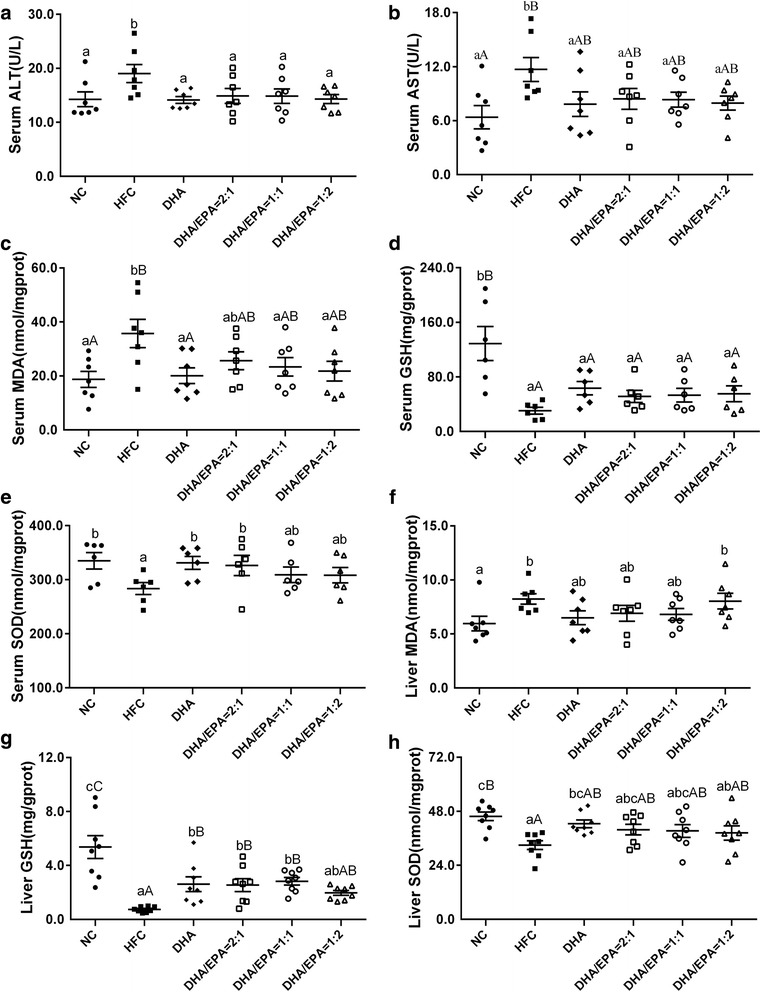
Effects of various ratios of DHA/EPA on liver damage in high-fat diet-induced mice. Serum ALT (**a**), AST (**b**), MDA (**c**), GSH (**d**), SOD (**e**), and liver MDA (**f**), GSH (**g**), SOD (**h**) levels were measured with the corresponding commercial kits. Each value is the mean ± SEM (*n* = 6), and any two groups without the same lowercase or uppercase letters, as marked in the figures, indicate a significant difference of *p* < 0.05 and *p* < 0.01, respectively

Levels of serum ALT, AST, GSH, SOD, and MDA, and liver GSH, SOD, and MDA are presented in Fig. 3. The results show that treatment with n-3 PUFAs lowered the levels of serum ALT and AST and both serum and liver MDA production, and raised the levels of GSH and SOD both in serum and liver in comparison with those in the HFC group. Nonetheless, no significant differences were found in the levels of any of these biomarkers between the DHA and DHA/EPA groups. Nevertheless, supplementation with a DHA/EPA ratio of 1:2 resulted in greater reduction in serum ALT, AST, and MDA levels and increase of serum GSH levels among the DHA/EPA groups; however, this increase was not higher than that observed in DHA groups. Collectively, supplementation with a DHA/EPA ratio of 2:1 was the most effective among the three DHA/EPA groups at enhancing SOD levels both in serum and liver, but these levels were not higher than those in the DHA group. Particularly, mice treated at a DHA/EPA ratio of 1:1 showed increased liver GSH levels as compared to the DHA group (2.828 ± 0.279 vs. 2.612 ± 0.546 mg/g prot).

### Effects of various ratios of DHA/EPA with an n-6/n-3 ratio of 4:1 on lipid metabolism genes

**Fig. 4:**
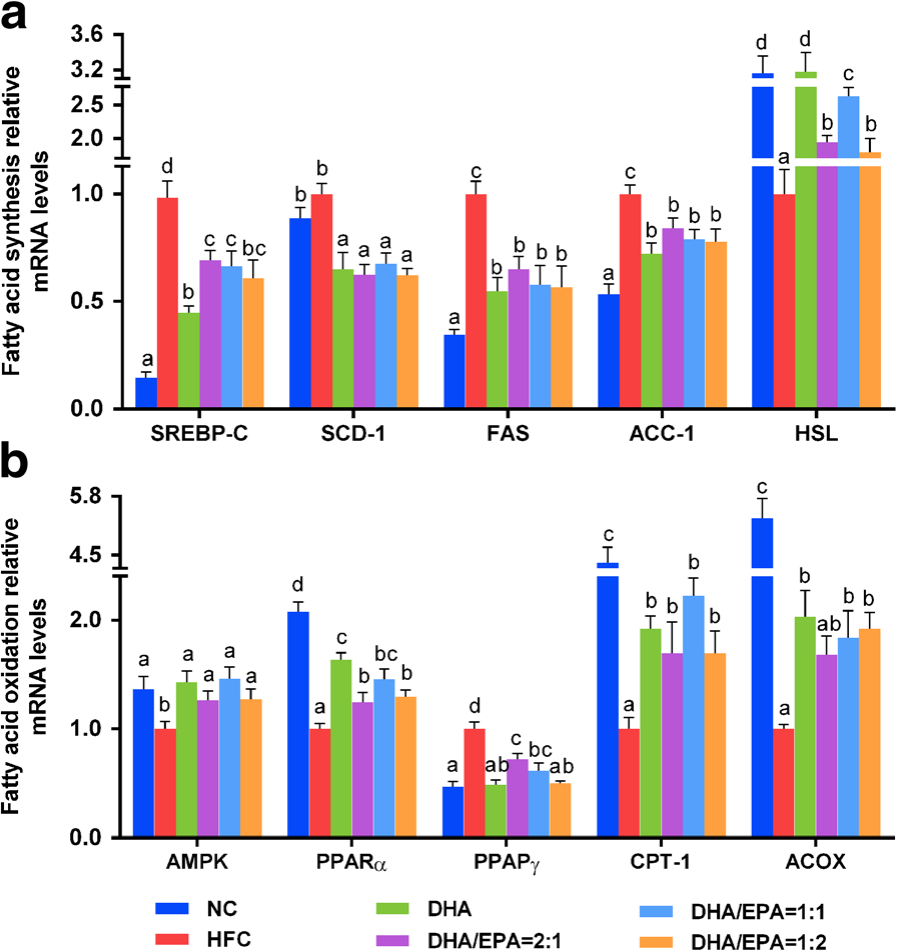
Effects of DHA/EPA on relative lipid metabolic gene expression in high-fat diet-induced mice. The relative mRNA expression of genes involved in fatty acid synthesis (**a**) and fatty acid oxidation (**b**) in liver tissues analyzed by real-time PCR. Each value is the mean ± SEM (*n* = 8), any two groups without the same lowercase or uppercase letters, as marked in the figures, indicate a significant difference of *p* < 0.05 and *p* < 0.01, respectively

Gene expression variation of the enzymes and transcription factors of lipid metabolism was measured by real-time PCR as shown in Fig. 4. Compared to the HFC group, 43%, 27%, 46%, and 27% increases in AMPK (AMP-activated protein kinase) mRNA expression, and 103%, 68%, 84%, and 92% increases in ACOX (acyl-coenzyme A oxidase) mRNA expression, were demonstrated in DHA groups and groups with the DHA/EPA ratio of 2:1, 1:1, and 1:2, respectively. Noticeably, mice treated with a DHA/EPA ratio of 1:2 showed a greater reduction in SREBP-1C (sterol regulatory element binding protein-1C), SCD-1 (stearoyl-CoA desaturase-1), FAS (fatty acid synthase), ACC-1 (acetyl CoA carboxylase-1), and PPARγ (peroxisome proliferator activated receptor gamma) mRNA expression, while the DHA/EPA 1:1 group showed higher HSL (hormone sensitive lipase), PPARα, and CPT-1 (carnitine palmitoyl transferase-1) mRNA expression, among the DHA/EPA groups. Particularly, the group treated with the DHA/EPA ratio of 1:2 showed lowered SCD-1 mRNA expression, and the DHA/EPA 1:1 group showed enhanced CPT-1 mRNA expression, whereas no significant differences were observed in the DHA group.

### Effects of various ratios of DHA/EPA with an n-6/n-3 ratio of 4:1 on the inflammatory response

**Fig. 5:**
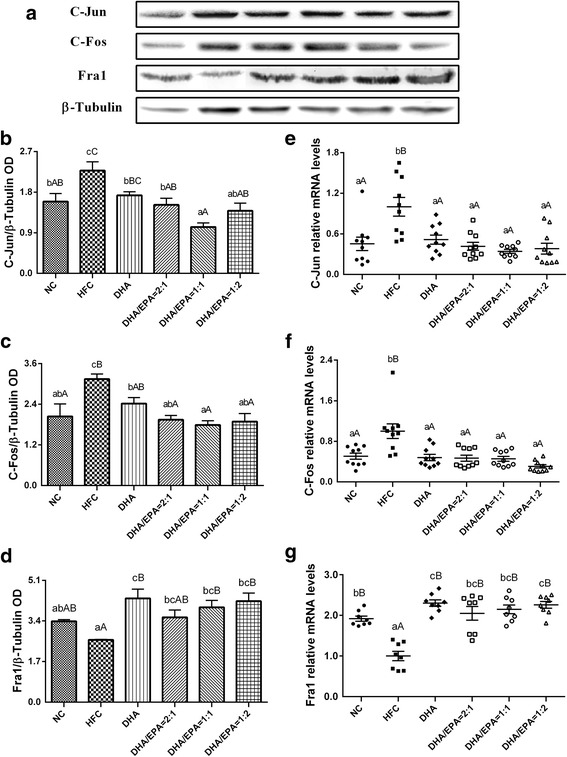
Effects of various ratios of DHA/EPA on relative protein expression in high-fat diet-induced mice. **a** Bands of c-Jun, c-Fos, and Fra1 obtained by western blotting. The western blot results of c-Jun (**b**), c-Fos (**c**), and Fra1 (**d**) in liver tissues. The mRNA expression of c-Jun (**e**), c-Fos (**f**), Fra1 (**g**) in liver tissues as detected by real-time PCR. Each value is the mean ± SEM (*n* = 3), and any two groups without the same lowercase or uppercase letters, as marked in the figures, indicate a significant difference of *p* < 0.05 and *p* < 0.01, respectively

The protein expression levels of the three main subunits of AP-1 (c-Jun, c-Fos, and Fra1) were detected by western blot (Fig. 5a–d). The protein expression levels of c-Jun and c-Fos were significantly lower in the DHA/EPA groups compared to the HFC group (*p* < 0.01), meanwhile supplementation with a DHA/EPA ratio of 1:1 revealed greater reductions in the expression levels of these two proteins in contrast to the DHA group. Nonetheless, the protein expression levels of Fra1 were found to be significantly higher in the DHA and DHA/EPA treated groups in comparison to the HFC group (*p* < 0.05). Supplementation with DHA/EPA at the ratio of 1:2 did not result in enhancement in Fra1 expression levels. The mRNA expression levels related to c-Jun, c-Fos, and Fra1 were quantified by real-time PCR (Fig. 5e–g). Supplementation with n-3 PUFA showed a significant decrease in c-Jun and c-Fos mRNA expression and an increase in Fra1 mRNA expression, as compared to those in the HFC group (*p* < 0.05). Moreover, mice treated with the DHA/EPA ratio of 1:1 and 1:2 showed lowered c-Jun and c-Fos mRNA expression, respectively, in comparison to those in the DHA group. Nevertheless, no significant increase in Fra1 mRNA expression was observed in the best performance group (treated with the DHA/EPA ratio of 1:2) as compared with the DHA group.

**Fig. 6:**
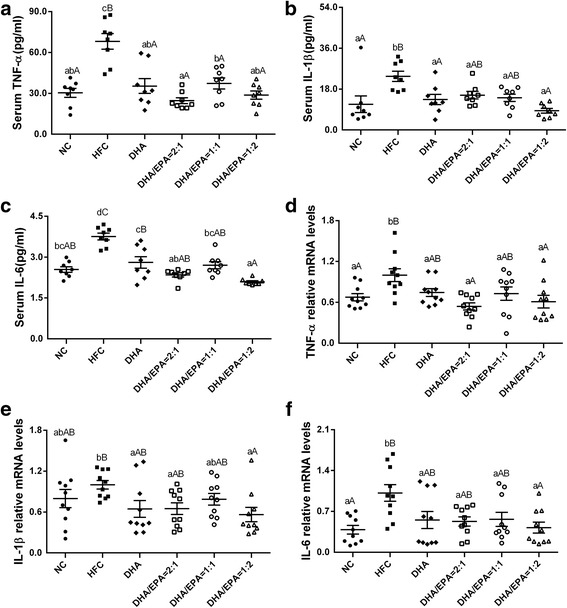
Effects of various ratios of DHA/EPA on inflammatory cytokines in high-fat diet-induced mice. Serum levels of TNF-α (**a**), IL-1β (**b**), and IL-6 (**c**) as measured by ELISA (*n* = 8). The expression of TNF-α (**d**), IL-1β (**e**), IL-6 (**f**) in hepatic tissues as measured by real-time PCR (*n* = 10). Each value is the mean ± SEM, and any two groups without the same lowercase or uppercase letters, as marked in the figures, indicate a significant difference of *p* < 0.05 and *p* < 0.01, respectively

The concentrations of serum TNF-α, IL-1β, and IL-6 were all significantly lower in the DHA and the DHA/EPA groups as compared to those in HFC group (*p* < 0.05) (Fig. 6). The quantitative real-time PCR assay, which roughly identified the inflammatory cytokine levels, revealed significantly decreased expression of TNF-α, IL-1β, and IL-6 in liver tissues of DHA/EPA-treated mice in comparison to those in HFC-treated mice (*p* < 0.05). Furthermore, mice treated with a DHA/EPA ratio of 2:1 revealed greater decreases in serum TNF-α contents and its mRNA expression levels in contrast to the DHA group. Additionally, supplementation with a DHA/EPA ratio of 1:2 demonstrated a more obvious reduction in serum IL-1β and IL-6 contents, and their mRNA expression as compared to those in the DHA group.

## Discussion

The n-3 PUFAs, principally DHA and EPA, have been reported to play an increasingly significant role in the growth and health of the human body as shown by many previous studies [9, 10]. The physiological functions of n-3 PUFAs have attracted increasing interest because of the increasing number of people choosing to lead healthy lifestyles [18]. However, there was a tendency among most attempted studies to focus on the effects of an individual n-3 or n-6 PUFA or the ratio of n-6/n-3, which did not take the possible influence of the relative ratio of DHA and EPA on the results into account [15]. A marked increase in the n-6/n-3 ratio is associated with lipogenesis, overweight lipid oxidation and secretion, and therefore promotion of NAFLD [11]; fish oil intake varies in its hepatic fatty acid composition in supplements with n-3 PUFA, and a reduction in the n-6/n-3 ratio has already been revealed in another study [7]. Other reports demonstrated that DHA supplementation led to a greater reduction in specific makers of inflammation than that by an equal dose of EPA [17], but supplementation with varying ratios of EPA/DHA influenced different metabolic syndrome markers [15] and enhanced the positive effects of biomarkers of inflammation [12]. Accordingly, in the present study, we concentrated on the effects of various ratios of DHA/EPA (with an n-6/n-3 ratio of 4:1) on liver damage in C57BL/6 J mice with high-fat diet-induced obesity.

High-fat diets enriched in saturated fat may promote an increase in body weight and lead to obesity, liver injury, hepatic insulin resistance, and steatosis [19]. Treatment with increasing amounts of n-3 PUFA, however, seems to reduce the rate of increase in body weight [20]. A study showed that animals on high-fat diets appeared to have an increased ability to store TG, whereas animals fed high fish oil diets (along with consumption of excess calories) did not convert excess lipids into TGs [19]. These data appear to be in agreement with our current study showing that lower TG levels both in serum and in the liver were found in groups that were treated with n-3 PUFAs compared with the HFC group. Meanwhile, all n-3 supplementation groups showed attenuation of body weight gain compared to that in the untreated control. EPA and DHA oil reduced total body fat [21], wherein reduced hepatic lipid deposition was observed in n-3 groups in the present study. Our results revealed that DHA and DHA/EPA supplementation decreased hepatic TG and TC levels possibly through reducing the release of TG and TC into the blood plasma for liver tissues, simultaneously improving serum HDL-C levels. Therefore, DHA and DHA/EPA supplementation inhibited hepatic lipid profile deterioration in mice with high-fat diet-induced obesity. Additionally, groups with a DHA/EPA ratio of 1:2 with an n-6/n-3 ratio of 4:1 showed the lowest serum levels of TG, TC, and LDL-C, and the highest concentration of serum HDL-C, while the lowest liver TC and TG levels were observed in the DHA/EPA 2:1 group among the DHA/EPA groups. Nevertheless, the best performance groups treated with DHA/EPA did not show better results than those in the DHA group. These findings seemed to contradict our previous report that indicated that serum TC and LDL-C levels were the lowest in the 2:1 DHA/EPA group, because the highest serum HDL-C content was observed in the 1:1 DHA/EPA group in *apoE* ^−/−^ mice [16]. These findings may be explained by the lack of apoE, a ligand for receptors that clear remnants of chylomicrons and very low density lipoproteins, and accumulation of apoE is expected to cause accumulation in plasma of cholesterol-rich residues [22].

The n-3 PUFAs exerted their effects on the lipid profiles through their abilities to upregulate genes encoding proteins involved in fatty acid oxidation, while concurrently downregulating genes encoding proteins involved in lipid synthesis [15]. For example, n-3 PUFAs downregulate the transcription factor SREBP-1 [23, 24], which upregulates lipogenic genes, FAS and SCD-1, promoting TG accumulation in the liver [25]. However, little was previously known about how different ratios of DHA/EPA affect various pathways involved in lipid metabolism in the liver. Thus, in order to understand the mechanism of action underlying the differences in lipid profile and liver lipid deposition caused by DHA/EPA supplementation, the mRNA expression levels of the following proteins were analyzed: SREBP-1C [26], SCD-1 [27], FAS [28], ACC-1 [4], and HSL [29], which are proteins involved in lipogenesis; and AMPK [30], PPARα [31], PPARγ [31], CPT-1 [32], and ACOX [8], which are proteins involved in fatty acid oxidation. Mice treated with DHA/EPA showed promotion of the expression of Fra1 protein and its relative mRNA expression, and inhibition of the expression of PPARγ mRNA, which potentially indicated that DHA/EPA exert their effects on lipid metabolic modulation by accelerating the expression of Fra1 and restraining the expression of PPARγ, and thereby suppressing the expression of their downstream adipogenic genes. Moreover, a DHA/EPA ratio of 1:1 revealed the highest expression of enzymes such as AMPK, PPARα, and CPT-1 relative mRNA levels, therefore improving fatty acid oxidation in the liver. The lowest relative mRNA expression levels of SREBP-1C, SCD-1, FAS, and ACC-1 were observed in the DHA/EPA 1:2 group, which indicated that supplementation with DHA/EPA is likely to alter fatty acid synthesis via upregulating AMPK expression, which decreases the expression of SREBP-1C and PPARγ, thereby reducing downstream expression of key enzymes (ACC-1, FAS, and SCD-1) in the liver. Consequently, the central protein that balances energy metabolism in the body, Fra1, plays a vital role in lipid metabolism by inhibiting the gene expression of PPARα, reducing SCD-1 levels, and decreasing de novo lipogenesis, which lowers hepatic lipid accumulation and lipogenesis [33].

Overnutrition results in the onset of oxidative stress in the liver because of higher availability and oxidation of fatty acids [34], and high-fat diets increase hepatic steatosis and hepatitis attributed to increased oxidative stress [35]. Additionally, lowered activation of GSH and SOD and increased MDA production both in serum and liver were observed in the HFC group compared to the NC group in the present study. Furthermore, evidence of decreased lipid content was found in animal models treated with n-3 PUFAs [36], whereby the improvement in lipid profile was principally caused by increased fatty acid β-oxidation and suppression of fatty acid synthesis in the liver by PUFAs [37]. MDA production was remarkably decreased by PUFA supplementation, and the inhibitory effects of mixed plant oils and DHA/EPA on MDA production were shown to be significantly different [16]. In the present study, we observed increased activation of GSH and SOD, which may explain the higher antioxidant capacity [14], and decreased MDA production was observed both in serum and in the liver in the DHA/EPA groups in comparison with that in the HFC group. Furthermore, the effects of various DHA/EPA ratios with a balanced n-6/n-3 ratio of 4:1 on liver damage biomarkers were investigated here, and lowered concentrations of serum ALT and AST were found. Nonetheless, a stronger effect was not observed in the best performance group among DHA/EPA groups as compared with the DHA group in mice with high-fat diet-induced obesity. Accordingly, the ability of DHA/EPA with an n-6/n-3 ratio of 4:1 to modulate the alteration in lipid oxidation and hepatic oxidative damage was substantial; this result is in line with an existing report showing that supplementation with PUFAs induced changes in the oxidation state [16].

NAFLD, which ranges from simple hepatic steatosis to steatohepatitis, can be complicated with inflammation [34]. In NAFLD, cholesterol deposited in cytoplasmic droplets stimulates the secretion of pro-inflammatory factors, which amplifies the local inflammatory reaction and causes the production of ROS [16]. Regular intake of n-3 PUFA, mainly EPA and DHA, provides metabolic health benefits by altering the production of specific lipid biomarkers of cellular inflammation [38]. This observation is in agreement with the current study, which indicated that DHA/EPA supplementation attenuates inflammation by reducing levels of inflammatory risk factors. Although it has been proposed that DHA is more beneficial in terms of its anti-inflammatory effects as shown in various cellular models [39], it has been reported that supplementation with EPA/DHA at ratios of 1:1 and 2:1 reduces the inflammatory C reactive protein index more significantly than the ratio of 1:2 [13]. In the present study, levels of inflammatory factors as well as those of their relative gene expression decreased obviously; the biggest reduction in TNF-α levels was found in the DHA/EPA 2:1 group, while the lowest levels of IL-1β and IL-6 were observed in the DHA/EPA 1:2 group with an n-6/n-3 ratio of 4:1. This result is consistent with the observation of inflammation-dampening effects of n-3 PUFAs in the liver and a decreased inflammatory response in fat-1 mice that is associated with significantly reduced hepatic gene expression of TNF-α, IL-1β, and IL-6 [40].

Cytokines involved in the complex inflammatory response network can be modulated by various activators and inhibitors, thus, activating various reactions in inflammatory pathways [41], which may amplify the inflammatory reaction and result in tissue injury if uncontrolled [42]. Among the typical inflammatory responses of metaflammation, activating protein-1 (AP-1, including c-Jun and c-Fos) [43] is a relevant type of inflammatory transcription factor [44] and an important signal transduction pathway component of proinflammatory mediator expression that is independent of NF-κB [31]; its activity might be regulated by gene transcription levels and protein concentration [45]. The results of our present study suggest that consumption of DHA/EPA significantly suppressed the expression of c-Jun and c-Fos proteins and their respective genes, with ratios of 1:1 and 1:2 showing better effects than those by DHA, in HFD-induced mice. Consequently, DHA/EPA reduced the expression of c-Jun and c-Fos proteins and weakened the activity of AP-1, which may decrease the expression of inflammatory factors; thus, this may have attenuated the activation of inflammatory pathways as a lower DHA/EPA ratio was found to be more effective at alleviating an inflammatory response.

## Conclusions

In summary, supplementation with various DHA/EPA ratios composed of fish, algae, and sunflower seed oils with an n-6/n-3 ratio of 4:1 was beneficial by means of promoting lipid metabolic processes, attenuating steatosis, and hepatic lipid accumulation, and by relieving hepatocyte oxidative damage by regulating the serum lipid profiles, controlling inflammatory reactions, and balancing lipid peroxidation. These effects of n-3 PUFA on lipid metabolism may be linked to the improvement of Fra1 expression and the attenuated activity of c-Jun and c-Fos, ultimately reducing the severity of a lipid metabolism disorder and liver damage to some extent. DHA/EPA did not yield better results on modulation of the serum lipid profile, oxidative damage, and expression of lipid metabolism-related genes, but were more effective at controlling inflammatory factors as compared to that by DHA. A lower DHA/EPA ratio seems to be more beneficial for alleviation of high-fat diet-induced liver damage in mice, and a DHA/EPA ratio of 1:2 mitigated the inflammatory risk factors. Further research is required to explain this discrepancy.

## Abbreviations

ACC-1: Acetyl CoA carboxylase-1
ACOX: Acyl-coenzyme A oxidase
ALA: α-linolenic acid
ALT: Alanine aminotransferase
AMPK: AMP-activated protein kinase
AP-1: Activator protein 1
AST: Aspartate aminotransferase
CPT-1: Carnitine palmitoyl transferase-1
DHA: Docosahexaenoic acid
EPA: Eicosapentaenoic acid
FAS: Fatty acid synthase
GSH: Glutathione
HDL-C: High-density lipoprotein-cholesterol
HFC: High-fat control group
HFD I: High fat diet I
HFD II: High fat diet II
HSL: Hormone sensitive lipase
IL-1β: Interleukin-1β
IL-6: Interleukin-6
LDL-C: Low-density lipoprotein-cholesterol
MDA: Malondialdehyde
NAFLD: Non-alcoholic fatty liver disease
NC: Normal control group
PPARα: Peroxisome proliferator activated receptor alpha
PPARγ: Peroxisome proliferator activated receptor gamma
PUFA: Polyunsaturated fatty acid
SCD-1: Stearoyl-CoA desaturase-1
SFA: Saturated fatty acid
SOD: Superoxide dismutase
SREBP-1C: sterol regulatory element binding protein-1C
TC: Total cholesterol
TG: Triglycerides
TNF-α: tumor necrosis factor-alpha
